# A Review of the Biological Activity and Structure–Property Relationships of the Main Compounds from *Schisandra chinensis*

**DOI:** 10.3390/nu17030436

**Published:** 2025-01-25

**Authors:** Bartosz Skalski, Elżbieta Kuźniak, Iwona Kowalska, Monika Sikora, Beata Olas

**Affiliations:** 1 Department of Plant Physiology and Biochemistry, Faculty of Biology and Environmental Protection, University of Łódź, 90-237 Łódź, Poland; elzbieta.kuzniak@biol.uni.lodz.pl; 2 Department of Phytochemistry, Institute of Soil Science and Plant Cultivation-State Research Institute, Czartoryskich 8, 24-100 Pulawy, Poland; ikowalska@iung.pulawy.pl; 3 Łukasiewicz Research Network, Łódź Institute of Technology, 90-570 Łódź, Poland; monika.sikora@lit.lukasiewicz.gov.pl; 4 Department of General Biochemistry, Faculty of Biology and Environmental Protection, University of Łódź, 90-236 Łódź, Poland; beata.olas@biol.uni.lodz.pl

**Keywords:** *Schisandra chinensis*, schisandrin, gomisin, antioxidant activity, anticancer properties, antiviral properties, liver protection, cognitive functions

## Abstract

*Schisandra chinensis* is a plant from the *Schisandraceae* family that grows in humid climates, such as forests and mountain slopes. This plant is attracting the attention of an increasing number of scientists around the world, mainly due to its medicinal properties. It contains a variety of bioactive compounds that exhibit significant biological activities, including lignans, flavonoids, phenolic acids, triterpenoids, organic acids and essential oils. This publication is a review of the latest knowledge and research conducted in the field of analysis of biologically active compounds isolated from *Schisandra chinensis*.

## 1. Introduction

*Schisandra chinensis* (Chinese magnolia vine, five-flavour berry) originates from East Asia, especially from areas of China and Russia. It is a vine from the *Schisandraceae* family that grows in forests, on mountain slopes and in areas with a humid climate. *S. chinensis* is valued not only for its beautiful red berries but also for its medicinal and adaptogenic properties and has been used in traditional Chinese medicine for centuries. Its berries are considered an adaptogen, a substance that helps the body adapt to various stress conditions. In addition, *S. chinensis* is used to improve liver function, strengthen the immune system, improve physical and mental conditions and regulate hormonal functions. Nowadays, *S. chinensis* is gaining increasing popularity as a dietary supplement and ingredient in many health products. Its extracts are added to drinks, supplements and cosmetic preparations given their nutritional and skin-protective properties. *Schisandra* berries have a sour taste, which gives them their characteristic aroma, and they can be eaten raw, dried or processed in the form of syrups, juices, teas or even vodka. In traditional cuisine, these berries are also used to flavour dishes and drinks [1,2].

Due to the significant biological activity of active compounds in living organisms, it is important to sufficiently research and analyse the biological processes in the body caused by the activity of these active compounds, as well as examine the relationship between their internal structure and their biological properties. These components, such as lignans, flavonoids, organic acids and essential oils, are responsible for the various health benefits associated with this plant. *S. chinensis* fruits have been used in traditional Chinese medicine for 1000 years [3]. Lignans such as schisandrin and gomisin are recognised as the main bioactive compounds in *S. chinensis*. The focus of the research is on their impact on the immune system, liver function, hormonal regulation and antioxidant properties. Knowledge and analysis of the chemical structure and mechanisms of action are crucial to understanding the effectiveness of *Schisandra* as an adaptogen and plant medicine. Additionally, examining the relationship between the chemical structure and biological activity allows for a better contribution to the beneficial effects of *S. chinensis* on human health. This may lead to the development of more effective medicinal preparations and dietary supplements based on this plant. Interest in the health-promoting potential and properties of *S. chinensis* creates an opportunity to develop new treatment strategies in modern therapy and to promote the use of natural health-promoting compounds in medicine [2].

## 2. Botanical Overview of *S. chinensis*

*S. chinensis* belongs to the small family *Schisandraceae* of the order *Austrobaileyales*. The family *Schisandraceae* comprises approximately 90 species distributed mainly in temperate and subtropical forests in Southeast Asia and North America. Phylogenomic analysis showed that *Schisandraceae* is a sister to the clade that includes magnoliids, monocots and eudicots within angiosperms and was one of the earliest diverging angiosperm lineages [4]. *Schisandraceae* consists of three genera: *Schisandra Michx*, *Kadsura* Kaempf. ex Juss. and *Illicium* L. The genus *Schisandra* contains approximately 25 species (*S. arisanensis*, *S. bicolor*, *S. chinensis*, *S. elongata*, *S. glabra*, *S. glaucescens*, *S. grandiflora*, *S. henryi*, *S. incarnata*, *S. lancifolia*, *S. longipes*, *S. macrocarpa*, *S. micrantha*, *S. neglecta*, *S. parapropinqua*, *S. perulata*, *S. plena*, *S. propinqua*, *S. pubescens*, *S. pubinervis*, *S. repanda*, *S. rubriflora*, *S. sphaerandra*, *S. sphenanthera*, *S. tomentella*). Most biologically active compounds are found mainly in fruits, leaves and stems; unfortunately, there is still a lack of sufficient literature data that would provide a broader description of research on individual species. Literature sources refer to only a few species, but due to its most widely described and best-researched biological activity, *S. chinensis* is the most popular *Schsandra* plant used in traditional Chinese natural medicine. *S. chinensis*, the dried ripe fruits is the best-known representative of this genus and described in more detail in this paper. *S. chinensis* is native to Russia, north-eastern China, Japan and Korea. In Russia, it grows naturally in the regions of Primorsk and Khabarovsk, the Kuril Islands and southern Sacchalin [5]. It is cultivated mainly in China and the Republic of Korea, as well as in North America and Europe, as an ornamental plant for its fragrant white to pink flowers and attractive fruits. In rural Northeast China, *S. chinensis* holds significant economic value as a unique cash crop. It is used there as a traditional tonic herb and food, with an annual demand of more than 30,000 tons [6].

*S. chinensis* occurrence is influenced by ecological factors such as climate and soil characteristics. Suitable habitats are characterised by a humid climate, an average growing-season temperature of 18.69–20.99 °C, moderate light and wet, humus-rich soil with a slightly acidic pH [6].

*S. chinensis* is a deciduous woody climbing vine growing from 0.5 to 25 m tall [6]. It lacks tendrils and other specialised structures for climbing, but due to spiral stem growth, it wraps around the tree trunks and becomes entangled with any neighbouring objects. It forms short lateral shoots that develop from the previous year’s growth. Bracts at the base of these lateral shoots protect the vegetative shoot apex before shoot elongation. The plant produces roots from nodes in prostrate stems [7]. The bark of young shoots is red-brown and glossy before turning reddish brown. The leaves with red petioles up to 3 cm long are arranged in bunches and are 5–11 cm long, 2–7 cm wide, ovate or oblong-obovoid, apex acute or acuminate, base cuneate or broadly cuneate [5]. The leaves are dark green and glossy, with slightly pubescent veins on the abaxial lamina surface, and hypostomatous [7]. Flowers are unisexual, dioecious, solitary or clustered axillary, yellowish-white to pale pink and fragrant. The diameter is up to 2 cm with a simple spirally arranged perianth. An average of 3–5 flowers grow from the leaf axils on hanging stalks up to 4 cm long. The perianth comprises 6–9 segments [8]. Male flowers are stalked, with five stamens and filaments united into a short column, the female flowers with numerous carpels [5]. Due to the low female-to-male flower ratio, the development and number of female flowers determine the yield of *S. chinensis* [9]. The fruits of *S. chinensis* are deep-red, globular, juicy berries reaching 5–8 mm in diameter and arranged into dense, grape-like bunches reaching a length of 6–10 cm. Each berry contains 1–2 yellowish-brown, shiny, kidney-shaped seeds [5,10]. All parts of the plant have an odour resembling a lemon smell. *S. chinensis* is an entomophilous plant species [8].

## 3. Identification and Classification of Key Active Compounds of *S. chinensis*

**Figure 1:**
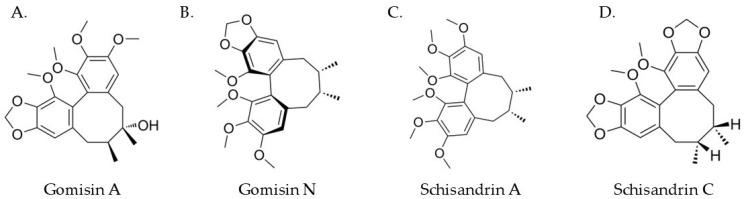
Chemical structures of selected lignans present in *S. chinensis*. (**A**) Gomisin A; (**B**) Gomisin N; (**C**) Schisandrin A; (**D**) Schisandrin C.

*S. chinensis* contains many bioactive compounds, including lignans, flavonoids, phenolic acids, triterpenoids, organic acids and essential oils. The main ingredients of *S. chinensis* including a variety of plant substances that give it its unique health properties are summarised in Table 1. The chemical structures of selected lignans present in *S. chinensis* are shown in Figure 1. One of the main ingredients of this plant are detailed below.

### 3.1. Lignans

Lignans are common components of *S. chinensis*, as secondary plant metabolites, formed by oxidative dimerisation of two phenylpropanoid units, connected by a β,β′-ether bond, and they constitute the basic chemical structure. They may differ in substituents and additional functional groups [2,4]. Lignans consist of eight subgroups (furofuran, furan, dibenzylbutane, dibenzylbutyrolactol, dibenzylbutyrolactones, aryltetralin, darylnaphthalene, and ibenzocyclooctadienes). Furan, dibenzocyclooctadiene, and dibenzylbutane lignans are divided into “lignans with C_9_ (*9*′) oxygen” and “lignans without C_9_ (*9*′) oxygen” [11]. Lignans occur in various parts of the plant, primarily in the fruit, but also in the seeds, shoots, and leaves. Schizandrin (syn. schisandrol A, wuweizisu A) is the dominant lignan of *S. chinensis* [1,12]. The fruits of *S. chinensis* are rich in lignans (over 30 lignans have been isolated, e.g., dibenzocyclooctadiene: gomisin A; N, schisandrin A, γ-schisandrin, schisandrin, and schisandrin C) [10,12,13]. Literature data report the identification of 11 bioactive lignans, i.e., schisandrin, schisandrin B (syn. gomisin N, γ-schisandrin, wuwezisu B), schisandrin C, gomisin A (syn. schisandrol B), schisantherin A (syn. gomisin C, schisandrer A), schisantherin B (syn. gomisin B, schisandrer B), gomisin J, gomisin G, angeloylgomisin H, schisanhenol (syn. gomisin K3), and deoxyschisandrin (syn. schisandrin A). Liu et al. (2022) discovered the presence of nine common lignans, including schizandrin A, schizandrin B, gomisin D, gomisin G, gomisin J, angeloylgomisin H, schizandrin B-C, and schizandrin A. In the study by Hu et al., 11 characteristic lignans (schizandrin, schizandrin B, angeloylgomisin H, gomisin G and J, schizandrin A and B, deoxyschizandrin, γ-schizandrin, schizandrin B and C) were described in 22 samples from different sources of *S. chinensis* [14,15]. In contrast, six biologically active lignans (schizandrin, gomisin A and N, deoxyschizandrin, γ-schizandrin, and wuweizisu C) were extracted from the stems [25]. The results of Yang and Yuan [2] reported the identification of 86 lignans from *S. chinensis*, among which dibenzocyclooctadiene lignans are the major bioactive components, known as “schisandrin lignans” [2,45]. The lignan content varied with respect to the occurrence in the different parts of the plant, with the total content being highest in the roots and lowest in the leaves. Delay in harvest time resulted in a decrease in the lignan content in *S. chinensis* [46]. The amount of schisandrin contained in the fruits ranged from 2.2 to 14.5 mg/g, gomisin N from 2.1 to 12.2 mg/g, and gomisin A from 0.9 to 9.8 mg/g [47]. Szopa et al. (2018) described 14 lignans contained in *S. chinensis* fruit extracts, the concentrations of individual compounds of which ranged from 2.6 to 166.8 mg/100 g DW (dry weight). The dominant metabolites were schisandrin (166.8 mg/100 g DW), γ-schizandrin (96.2 mg/100 g DW), gomisin A (72.4 mg/100 g DW), angeloylgomisin H (71.6 mg/100 g DW) and schizantherin B (56.8 mg/100 g DW). The total content of Schisandra lignans in fruit extracts was determined to be 646.0 mg/100 g DW. Extracts from *S. chinensis* leaves (14 lignans isolated) showed significantly lower concentrations than in fruits and ranged from 1.4 to 55.1 mg/100 g DW. The total content of Schisandra lignans in leaf extracts was 240.7 mg/100 g DW. The following were identified in the highest amounts: schizandrin (55.1 mg/100 g DW), angeloylgomisin H (31.4 mg/100 g DW), schizandrin D (31.3 mg/100 g DW) and γ-schizandrin (24.5 mg/100 g DW). The total amount of lignans in the stem and rhizome of *S. chinensis* bark during the flowering stage of the plant was 6–11%, while the content of schizandrin and schizandrol/gomisin A was 3–8%. Schizandrin, gomisin A, deoxyschisandrin, gomisin N and wuweizisu C were identified in the seeds, in amounts of 0.75–1.86, 0.13–0.90, 0.07–1.09, 0.24–1.49 and 0.01–0.34%, respectively [48,49]. The literature reports various analytical methods for the analysis of lignans in *S. chinensis*. An example is the isolation and separation of gomisin A and schizandrin from *S. chinensis* using preparative counter-current high-current chromatography (HSCCC) with a two-phase solvent system [50]. Among others, the reversed-phase high-performance liquid chromatography (RP-HPLC) method with diode array detection (DAD) was developed and validated. It is a fast and specific method for determining four common Schisandra lignans (schizandrin, schizandrol B, deoxychizandrin and γ-schizandrin) present, among others, in commercial dried aqueous extracts of *S. chinensis* [51].

Gnabre et al. (2010) showed that benzocyclooctadiene lignans present in *S. chinensis* fruits (gomisin-N and deoxyschizandrin) were isolated using reversed-phase liquid chromatography (RP) with automated injection. Lu et al. (2012) identified 11 lignans using RP-HPLC with UV detection [52,53]. An important step in the identification process is the purification of lignans. For this purpose, Huang et al. (2013) described the use of column fractionation and supercritical counter-solvent precipitation method to purify lignans isolated from *S. chinensis* fruits [54]. Literature data report another study on the extraction of schizandrin, schizanderin A and deoxyschizandrin from *S. chinensis* seeds using an aqueous two-phase system coupled with ultrasound [55]. Another method widely described in the literature is high-speed counter-current chromatography (HSCCC) used for the preparative separation and purification of deoxyschizandrin and γ-schizandrin from crude petroleum ether extracts of *S. chinensis* [56]. Using this method combined with preparative high-performance liquid chromatography (HPLC), Zhu et al. (2015) purified 6 lignans from *S. chinensis* stems [57]. Mocan et al. (2016), using LC-DAD/ESI-ToF-MS technique, identified 28 lignans from *S. chinensis* leaves, stems and fruits [20]. The UPLC-Q/TOF-MS method identified schizandrin, schizandrol B, deoxyschizandrin, gomisin H and schizandrin B contained in *S. chinensis* oils. These compounds are considered to be the main bioactive compounds present in *S. chinensis*, which exhibit important health-related medicinal properties [58]. Schizandrin and other lignans present in *S. chinensis* fruits have a beneficial effect on human health [59,60,61]. These compounds primarily exhibit high antioxidant, hepatoprotective, anticancer and anti-inflammatory activity, may have antiviral and cytotoxic effects on the human immunodeficiency virus (HIV), as well as a beneficial effect on general physical fitness and the central nervous system. *S. chinensis* and its fruits have been used in traditional natural medicine as a herbal medicine helpful in the treatment of liver problems, in combating chronic cough or chronic fatigue [16,17,62,63,64,65]. However, it has been shown that *S. chinensis* fruits may have toxic effects on aquatic plants (Lemna minor) and invertebrate zooplankton species (Daphnia magna and Thamnocephalus platyurus) [18,19].

### 3.2. Flavonoids

*S. chinensis* also contains flavonoids, which are secondary plant metabolites and occur in every part of the plant (e.g., fruits, seeds, flowers, roots, leaves, or even the lignified parts). Flavonoids in *S. chinensis* include various subclasses such as flavones, flavonols, flavanones and flavan-3-ols. The presence of flavonoids (rutin, hyperoside, isoquercitrin, and quercetin) in *S. chinensis* have been reported by Mocan et al. (2014). By using LC-DAD/ESI-ToF-MS, twenty phenolic compounds were identified in the leaves, stems and fruits of *S. chinensis* by Mocan et al. (2016) [20,66]. In addition, quercetin glycosides and chlorogenic acid isomers contribute to over 80% of the total antiradical activity. Flavonoids such as kaempferol-3-O-glucoside-7-O-rhamnoside (5.68 mg/g DW), kaempferol-3-O-glucoside (5.12 mg/g DW), quercetin-3-O-glucoside (3.68 mg/g DW), quercetin-3-O-galactoside (2.49 mg/g DW), quercetin-3-O-glucoside-7-O-rhamnoside (1.70 mg/g DW), kaempferol (0.63 mg/g DW), quercetin-3-O-xyloside (0.49 mg/g DW), kaempferol-3-O-arabinose (0.48 mg/g DW), quercetin-3-O-dirhamnoside (0.42 mg/g DW), rutin (0.38 mg/g DW) and quercetin (0.29 mg/g DW), in *S. chinensis* leaves, have been identified and quantified by Mocan et al. (2016) [20]. Szopa et al. (2017) showed that *S. chinensis* leaves also contain kaempferol, myricetin and quercetin 3-ramnoside (quercitrin). The fruits of *S. chinensis* also include cyanidin derivatives: cyanidin-glucoside, cyanidin-xylosylrutinoside, cyanidin-rutinoside and cyanidin-xylosylglucoside, which belong to the anthocyanins [10,21,22,23]. In another study, flavonoids found in *S. chinensis* fruits include isoquercetin, quercetin and its derivatives: quercetin 3-rutinoside (rutin) and quercetin 3-galactoside (hyperoside) [24]. In the results of Szopa et al. (2017), five flavonoids (myricetin, kaempferol, quercetin, quercitrin and rutin) in *S. chinensis* extracts from biomasses of studied in vitro cultivation systems and from parent plant material were quantified. The highest total amounts of flavonoids (29.36 mg/100 g DW) were found in extracts from the biomass of agar cultures collected after 30 days of cultivation. The main flavonoid in all the studied systems was quercitrin (max. 27.43 mg/100 g DW) [10].

### 3.3. Phenolic Acids

The main bioactive compounds of the *S. chinensis* fruits are lignans, while phenolic acids are predominant in the leaves. Among phenolic acids, cinnamic acid (3.54 mg/g DW), 5-O-caffeoylquinic acid (chlorogenic acid; 4.99 mg/g DW), 3-O-caffeoylquinic acid (neo-chlorogenic acid; 2.95 mg/g DW), trans-5-O-p-coumaroylquinic acid (1.05 mg/g DW), 3-O-p-coumaroylquinic acid (0.87 mg/g DW), protocatechuic acid (0.53 mg/g DW), cis-5-O-p-coumaroylquinic acid (0.41 mg/g DW), 4-O-p-coumaroylquinic acid (0.26 mg/g DW), 3-O-ferruloylquinic acid (0.19 mg/g DW) and 4-O-caffeoylquinic acid (crypto-chlorogenic acid; 0.15 mg/g DW) have been identified and quantified by Mocan et al. (2016), in the leaves [20]. Only protocatechuic acid (0.34 mg/g DW) was detected in the fruits. In other research, the yield of cinnamic acid extracted from the leaves of *S. chinensis* was 0.18% DW, and its concentration in the extract was nearly 5% by weight [25]. In methanolic extracts from lyophilised biomass in vitro cultures of *S. chinensis*, using the RP-HPLC-DAD method, phenolic acids were also identified by Szopa et al. (2017). Seven free phenolic acids (chlorogenic, gallic, p-hydroxybenzoic, protocatechuic, salicylic, syringic and vanilic acids) were identified and quantified. The highest total amounts of phenolic acids (71.48 mg/100 g DW) were found in extracts from the biomass of agar cultures harvested after 30 days of cultivation and in the leaves and fruits. The main metabolites in all the tested systems were protocatechuic acid (max. 35.69 mg/100 g DW) and chlorogenic acid (max. 13.05 mg/100 g DW) [10]. The contents of cinnamic acid and six free phenolic acids were determined in methanolic extracts from the biomass of *S. chinensis* at different stages of organogenesis and in extracts from the aerial parts of plants growing in vivo. The total content of phenolic acids in both types of in vitro cultures was higher than in leaves (4.55 mg/100 g DW) and fruit (55.73 mg/100 g DW) of plants seeded in vivo. Chlorogenic acid and salicylic acid were the main compounds identified in biomass extracts from shoot-differentiating callus cultures (22.60 and 21.17 mg/100 g DW, respectively) [26].

### 3.4. Triterpenoids

The triterpenoids are other important groups of biologically active substances isolated from *S. chinensis* [67]. Schinortriterpenoids are characteristic constituents of the species of the *Schisandraceae*, in addition to the different triterpenoids of the lanostane type and the cycloartane type, which are typical isolates [3]. Triterpenoids are isolated from various parts of the plant. Cycloartane-type triterpenoids (schinchinenlactone A-C, henrischinin C, schinchinenin G and H, henrischinin A-B, schinchinenin A-F) were identified in leaves and stems [27]. Schinortriterpenoids (2β-hydroxymicrandilactone C, wuweizidilactone J, K, L, M, O and P, propindilactone Q, A and B, preschisanartanin E, F, K, L, M and N, arisanlactone C, schicagenin A-C, schisdilactone J, isoschicagenin C, schisdilactone A-I, schinesdilactone A and B, schindilactone H-K, arisanlactone B) were detected in the leaves and stems of *S. chinensis* [28,29,30]. Schinortriterpenoids (wuweizidilactone I and schindilactone H) were identified in fruits [31]. Rattan stems contain wuweizidilactone S and schindilactone LM, while in the roots, schinchinelactone D was identified [32,33]. Due to the significant bioactive function of triterpenoids, they should be regarded as indicator constituents for the quality assurance of *Schisandraceae* plants, especially *S. chinensis*, whose fruits have been used in traditional Chinese medicine for 1000 years [3].

### 3.5. Organic Acids

*S. chinensis* contains approximately 18% various organic acids, primarily citric acid, which is the main source of the sour taste [34]. Organic acids contribute to the flavour and nutritional properties of *S. chinensis* berries and may benefit the body’s digestion and metabolism [35]. Using HPLC and other techniques, it was found that fresh and ripe fruits of *S. chinensis* contain 3.26% citric acid, 1.13% malic acid and 0.53% shikimic acid [36]. Using the UHPLC-Q-TOF-MS technique, citric acid, 6-methyl citrate and dimethyl citrate were identified in *S. chinensis* [37]. In other research, thirty-nine organic acids in different processed products of *S. chinensis* fructus were determined which were obtained by methyl esterification and then analysed by gas chromatography-mass spectrometry (GC-MS). The main organic acids were citric acid, trimethyl ester; hexadecanoic acid, methyl ester; 9-octadecenoic acid, methyl ester; 8,11-octadecadienoic acid, methyl ester; 3-acetoxy-3-hydroxy propionic acid, methyl ester; ethanedioic acid, dimethyl ester; pentanoic acid, 4-oxo-methyl ester; butanedioic acid, dimethyl ester; pentanedioic acid, 3-oxo-dimethyl ester; 2-propenoic acid, 3-(4-hydroxy-3-methoxyphenyl)-, methyl ester and tetracosanoic acid, methyl ester. The contents of levulinic acid (4-oxopentanoic acid) increased up to 2.3-fold after vinegar processing [38].

### 3.6. Essential Oils

The essential oils present in *S. chinensis* give it its characteristic flavour and aroma. These oils are found primarily in the fruit and may also have antibacterial and antiviral effects, which can contribute to overall immune health support [39]. Using GC-MS techniques, up to 2.02% of oil was obtained from the fruit and its composition was highly complex, with up to 32 components [40]. In the Chen et al. (2012) report, 40 components were identified in the essential oil of *S. chinensis* fruits, and the main components were ylangene (37.72%), β-himachalene (10.46%) and α-bergamotene (8.57%) [41]. The results of Teng and Lee (2014) revealed that the major ingredients in the oil extracted by simultaneous distillation extraction were ylangene (15.01%), α-phellandrene (8.23%), β-himachalene (6.95%) and cuparene (6.74%) [42]. Li et al. (2003) studied the composition of the essential oil of *S. chinensis* derived from steam distillation and characterised 48 various volatile components of the oils. It was proven that the constituents of *S. chinensis* seed essential oil were separated and identified by GC-MS [43]. Copaene, α-farnesene and α-cubebene were found to be the main constituents of the essential oil from the seeds. Some components such as δ-selinene, germacrene B and germacrene D were also reported [44].

Taken together, the main ingredients of *S. chinensis* form a comprehensive blend of substances with potential health benefits that are used in both traditional medicine and modern health preparations.

## 4. Overview of Studies Demonstrating the Biological Effects of *S. chinensis*

### 4.1. Key Antioxidant Components and Their Mechanisms of Action

*S. chinensis*, a plant renowned for its medicinal properties, contains a variety of bioactive compounds that exhibit significant antioxidant activities. The primary lignans present in *S. chinensis*, such as schisandrin, schisandrin B, schisantherin and gomisin A, play crucial roles in mitigating oxidative stress by scavenging free radicals and enhancing the activities of endogenous antioxidant enzymes, namely superoxide dismutase (SOD) and glutathione peroxidase (GPx) [68]. These lignans help protect cellular components from oxidative damage, thereby maintaining cellular integrity and function. Additionally, the leaves and fruits of *S. chinensis* are rich in flavonoids, including isoquercitrin, quercetin and rutin, which are known for their potent antioxidant properties. Comparative studies have shown that the leaves possess a higher flavonoid content and greater antioxidant activity in vitro than the fruits. This increased antioxidant capacity is attributed to the higher concentration of flavonoids in the leaves, which effectively scavenge free radicals and inhibit lipid peroxidation [24,69]. Recent studies have expanded the understanding of the mechanisms through which *S. chinensis* exerts its antioxidant effects. A study by Gao et al. demonstrated that *S. chinensis* extract could upregulate the expression of nuclear factor erythroid 2–related factor 2 (Nrf2), a key regulator of the cellular antioxidant response. By activating the Nrf2 pathway, *S. chinensis* enhances the production of endogenous antioxidants, thus providing a robust defence against oxidative stress [70]. Moreover, research by Wang et al. revealed that *S. chinensis* lignans could mitigate oxidative damage in neuronal cells by reducing the production of reactive oxygen species (ROS) and improving mitochondrial function. This neuroprotective effect is particularly significant in the context of neurodegenerative diseases, where oxidative stress plays a critical role in disease progression [71]. In addition to its direct antioxidant effects, *S. chinensis* also modulates signalling pathways involved in inflammation and cell survival. A study by Zhang et al. highlighted that *S. chinensis* extracts could inhibit the activation of the nuclear factor kappa-light-chain-enhancer of activated B cells (NF-κB) pathway, thereby reducing inflammation and enhancing cell survival under oxidative stress conditions [72]. Furthermore, clinical studies have begun to explore the therapeutic potential of *S. chinensis* in various oxidative stress-related conditions. A randomised controlled trial conducted by Li et al. found that supplementation with *S. chinensis* extract improved antioxidant enzyme activities and reduced markers of oxidative stress in patients with metabolic syndrome, suggesting potential benefits for managing conditions associated with chronic oxidative stress. Overall, the comprehensive antioxidant effects of *S. chinensis* are mediated through a combination of direct free radical scavenging and the modulation of cellular antioxidant defence mechanisms. These multifaceted actions underscore the therapeutic potential of *S. chinensis* in protecting against oxidative stress and improving overall cellular health [73].

### 4.2. Antibacterial Properties

Recent studies have expanded the understanding of the antibacterial properties of *S. chinensis*. A study by Zhang et al., 2021, investigated the antimicrobial mechanisms of *S. chinensis* extracts against methicillin-resistant *Staphylococcus aureus* (MRSA). The study found that the extracts not only inhibited bacterial growth but also disrupted biofilm formation, a critical factor in MRSA’s resistance and persistence [74]. Moreover, research by Kim et al. explored the antibacterial activity of *S. chinensis* against *Helicobacter pylori*, a bacterium associated with gastric ulcers and cancer. The study demonstrated that the plant extracts significantly inhibited the growth of *H. pylori* and reduced the expression of virulence factors, suggesting potential therapeutic applications in managing *H. pylori* infections [75]. In addition to whole extracts, efforts have been made to isolate specific compounds responsible for the antibacterial activity of *S. chinensis*. A notable study by Jeong et al., 2023, focused on isolating antibacterial compounds from *S. chinensis* effective against *Streptococcus mutans*, a primary bacterium responsible for dental caries. The 30% ethanol extract of the plant was particularly effective, and further fractionation revealed that the butanol fraction exhibited high antibacterial activity. Subsequent isolation identified tartaric acid as a significant active compound [76]. Further isolation and characterisation studies by Liu et al., 2022, identified gomisin A and schisandrin B as potent antibacterial agents against various Gram-positive and Gram-negative bacteria. These lignans were shown to disrupt bacterial cell membranes, leading to cell lysis and death [77]. Studies have also investigated the potential synergistic effects of *S. chinensis* extracts with conventional antibiotics. A study by Park et al., 2023, examined the combination of *S. chinensis* extract with ampicillin and found a synergistic effect against multidrug-resistant *Escherichia coli*. This combination therapy significantly reduced bacterial load compared to the antibiotic alone, suggesting that *S. chinensis* could enhance the efficacy of existing antibiotics and help combat antibiotic resistance [78]. The promising antibacterial properties of *S. chinensis* have led to explorations of its potential clinical applications. Clinical trials are being conducted to assess the effectiveness of *S. chinensis* extracts in treating infections in humans. Preliminary results from a trial by Lee et al. 2023, indicated that the topical application of *S. chinensis* extract could effectively reduce symptoms of bacterial skin infections, highlighting its potential as a natural antimicrobial agent for dermatological use [79].

### 4.3. Anticancer Activity

*S. chinensis* has demonstrated notable anticancer effects, primarily through mechanisms such as the induction of apoptosis, the inhibition of cell proliferation, the reduction in oxidative stress and the modulation of inflammation and critical signalling pathways. The lignans present in *S. chinensis*, such as schisandrin, schisandrin B, schisantherin and gomisin A, play pivotal roles in these processes [17]. One of the main mechanisms by which *S. chinensis* exerts its anticancer effects is through the induction of apoptosis in cancer cells. Apoptosis, or programmed cell death, is essential for eliminating cancerous cells. Studies have shown that lignans from *S. chinensis* can trigger apoptosis by activating various cellular pathways, including the mitochondrial pathway and death receptor pathways. For instance, a study by Jafernik et al. demonstrated that schisandrin B induces apoptosis in breast cancer cells by activating caspase-3 and -9, essential components of the mitochondrial apoptotic pathway [80]. Schisandra lignans have also been found to inhibit the proliferation of cancer cells by causing cell cycle arrest at different phases, thereby preventing cancer cells from multiplying. Research by Rybnikar et al. revealed that gomisin A induces G1 phase cell cycle arrest in colorectal cancer cells by downregulating cyclin D1, a key regulator of the cell cycle [81]. The antioxidant properties of *S. chinensis* contribute significantly to its anticancer effects. By reducing oxidative stress, which is a contributing factor to cancer development, *Schisandra* extracts help protect cells from DNA damage and other harmful effects of free radicals. Studies have shown that these antioxidant properties are mainly due to the high flavonoid content in *S. chinensis*, which effectively scavenges free radicals and enhances the activities of endogenous antioxidant enzymes such as superoxide dismutase (SOD) and glutathione peroxidase (GPx) [68,82]. Chronic inflammation is also a well-known risk factor for cancer development and progression. *Schisandra* lignans exhibit strong anti-inflammatory properties by inhibiting the NF-κB pathway, a key regulator of inflammatory responses. This reduction in inflammation can contribute to a decreased risk of cancer development. Research by Park et al. demonstrated that schisandrin A significantly reduces the expression of pro-inflammatory cytokines in lung cancer cells by inhibiting NF-κB activation [83]. *S. chinensis* also affects various signalling pathways involved in cancer progression. Notably, the lignans have been shown to modulate the PI3K/Akt/mTOR pathway, crucial for cell growth and survival. By inhibiting this pathway, *Schisandra* extracts can reduce tumour growth and enhance the effectiveness of other anticancer therapies. Choi et al. found that schisandrin B inhibits the PI3K/Akt/mTOR pathway in hepatocellular carcinoma cells, leading to reduced cell viability and increased sensitivity to chemotherapy [84]. Emerging research continues to explore the breadth of *S. chinensis* anticancer properties, as exemplified by a study by Li et al. which investigated the synergistic effects of *S. chinensis* extract with conventional chemotherapeutic agents. The study found that combining the *Schisandra* extract with doxorubicin significantly enhanced the drug’s efficacy against breast cancer cells, suggesting potential applications in combination therapy to reduce drug resistance and side effects [85]. Moreover, research by Wang et al. is examining the impact of *S. chinensis* on cancer stem cells, which are often responsible for cancer recurrence and metastasis. Preliminary findings indicate that Schisandra lignans can target and reduce the viability of cancer stem cells in pancreatic cancer, providing a potential strategy for preventing cancer relapse [63].

### 4.4. Liver Protection

*S. chinensis* modulates lipid metabolism effectively, particularly in the management of NAFLD. Recent studies have provided further insights into its mechanisms. Feng et al. found that polysaccharides from *Schisandra* reduced serum levels of triglycerides (TGs), total cholesterol (TC) and low-density lipoprotein cholesterol (LDL-C), while increasing high-density lipoprotein cholesterol (HDL-C). This lipid-regulating effect is achieved by downregulating sterol regulatory element-binding proteins (SREBPs) and upregulating genes involved in lipid oxidation and metabolism, such as PPARα and AMPK. Additionally, research by Liu et al. demonstrated that *Schisandra* lignans activate AMPK, leading to enhanced fatty acid oxidation and improved lipid profiles in animal models of NAFLD. This study highlighted the potential of *Schisandra* as a therapeutic agent for metabolic disorders related to lipid metabolism [9]. Moreover, the anti-inflammatory properties of *Schisandra* are well documented and are mediated through the inhibition of pro-inflammatory cytokines and pathways. Lignans such as gomisin N inhibit the activation of nuclear factor-kappa B (NF-κB) and reduce the expression of inflammatory mediators, thereby reducing inflammation and subsequent liver damage. Recent research by Zhang et al. confirmed these findings, showing that gomisin N significantly decreased the levels of TNF-α, IL-6, and IL-1β in a mouse model of liver inflammation. This anti-inflammatory action contributes to the overall hepatoprotective effects of *Schisandra* [72]. *S. chinensis* has also shown potential in preventing liver fibrosis, a severe condition resulting from chronic liver injury. The ethanolic extract of *Schisandra* berries (SBE) inhibits the activation of hepatic stellate cells (HSCs), key players in the development of fibrosis. By modulating the TGF-β signalling pathway and reducing the deposition of collagen, Schisandra helps maintain normal liver architecture and function. A study by Kim et al. found that SBE reduced liver fibrosis markers in a rat model, supporting the notion that *Schisandra* can prevent and potentially reverse liver fibrosis [47]. Additionally, *Schisandra* components induce the expression of cytochrome P450 enzymes, particularly CYP3A and CYP2E1, which are involved in the detoxification of various endogenous and exogenous compounds. This induction accelerates the metabolism and clearance of toxins from the liver, providing a protective effect against liver damage. Recent findings by Park et al. demonstrated that *Schisandra* extracts significantly increased CYP3A and CYP2E1 activity in human liver cell lines, enhancing the liver’s ability to detoxify harmful substances. *Schisandra* also promotes liver cell regeneration and repair. The hepatoprotective effect includes enhancing the proliferation of hepatocytes and reducing apoptosis. This regenerative capability aids in the recovery of liver function after injury. A study by Li et al. showed that *Schisandra* extracts promoted hepatocyte proliferation and inhibited apoptosis in a model of liver injury induced by carbon tetrachloride (CCl4). This finding underscores the potential of *Schisandra* in supporting liver regeneration and recovery [86].

### 4.5. Improved Cognitive Functions

Research suggests that *S. chinensis* can protect against neuronal damage and enhance cognitive functions. Studies indicate that its bioactive compounds, particularly lignans, possess antioxidative properties that help mitigate oxidative stress, a key factor in neurodegenerative diseases and cognitive decline [1]. Animal studies have demonstrated that *S. chinensis* extracts can improve memory and learning abilities. One study showed that rats administered with *S. chinensis* extract exhibited enhanced spatial memory and learning capabilities in maze tests, which are standard assessments for cognitive functions in animal models [1]. The cognitive benefits of *S. chinensis* are believed to be mediated through several mechanisms. These include the modulation of neurotransmitter levels, the enhancement of cholinergic function and the upregulation of neurotrophic factors such as brain-derived neurotrophic factor (BDNF), which plays a crucial role in neuron growth and synaptic plasticity [1]. While most of the evidence comes from animal studies, some human studies also support the cognitive benefits of *S. chinensis*. For example, traditional uses of *Schisandra* in Chinese medicine include improving mental clarity and reducing mental fatigue. Modern clinical trials, though limited, have reported improved cognitive performance and reduced symptoms of stress in individuals consuming *Schisandra* extracts [87].

## 5. Current Trends and Future Directions

**Figure 2:**
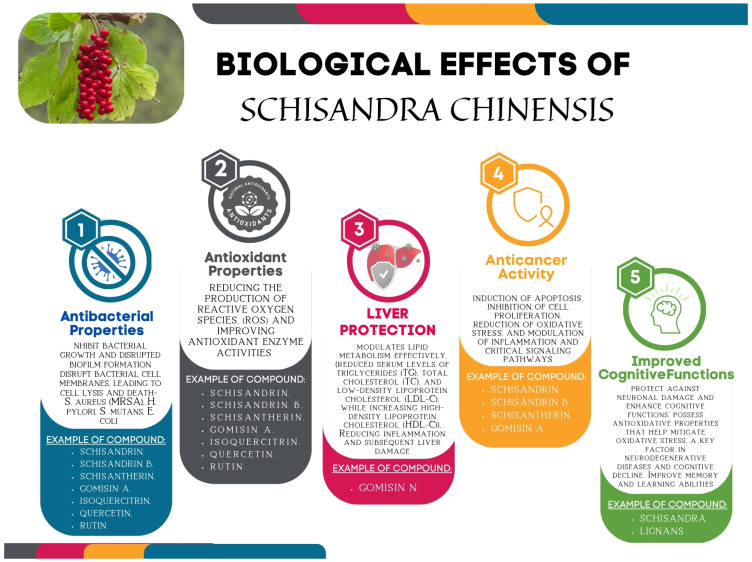
Biological activity of *S. chinensis*.

By analysing the available literature reports, it is possible to observe significant progress in analytical techniques, which undoubtedly have greatly facilitated the understanding of molecular mechanisms of compounds with the therapeutic potential from *S. chinensis*. Based on the reviewed literature, it can be concluded that *S. chinensis* lignans play a promising role as a natural bioactive compound, exerting neuroprotective effects by regulating various pathophysiological processes. The recognised chemical structure of these bioactive lignans may play a prominent role as active substances in new generations of drugs. However, although the recognised lignan extracts from *S. chinensis* in the described experimental in vitro and in vivo studies showed strong biological properties, clinical trials are still crucial and necessary. Recent research on *S. chinensis* has highlighted several advancements in understanding its bioactive compounds and their potential therapeutic benefits (Figure 2) [60].

## 6. Advanced Extraction and Analysis Techniques

New extraction methods and mass spectrometry technologies have significantly improved the detection and identification of *S. chinensis* components. Techniques such as UPLC-QTOF-MS (ultra-performance liquid chromatography coupled with quadrupole time-of-flight mass spectrometry) have been used to profile the metabolites of different parts of the plant, revealing a diverse array of bioactive compounds, including lignans, triterpenoids, flavonoids and tannins [69,77].

## 7. Neuroprotective and Cognitive Enhancements

*S. chinensis* has shown promise in enhancing cognitive functions and protecting against neurodegenerative diseases. Compounds such as schisandrin and schisandrin B have been found to reduce oxidative stress and improve memory and learning in animal models, suggesting potential benefits for a number of conditions including Alzheimer’s disease [77].

## 8. Metabolic and Cardiovascular Benefits

Recent studies have explored the effects of *S. chinensis* on metabolic health and cardiovascular diseases. Its bioactive compounds have been linked to improved lipid metabolism and reduced risk factors for heart disease. Additionally, Schisandra’s potential in regulating blood glucose levels and supporting overall metabolic health has been a focus of recent investigations [77].

## 9. Conclusions

The relationship between the chemical structure of *S. chinensis* compounds and their biological activity has been thoroughly investigated. Lignans such as schisandrin, gomisin and schisandrins A to E are the main components of *S. chinensis* and exhibit a wide range of biological activities. First of all, they have antioxidant properties that help remove free radicals and protect cells from oxidative damage. Some lignans also have a hepatoprotective effect, supporting the protection and regeneration of liver cells. Schisandrin B and schisantherin A also play an important role in the biological properties of *S. chinensis*. as they contain anti-inflammatory, anticancer and hepatoprotective properties [88]. Many studies have shown that triterpenoids can regulate liver enzyme activity and reduce liver inflammation. Polysaccharides, which are another important component of *S. chinensis*, have an immunomodulatory effect by increasing the activity of immune cells such as macrophages and lymphocytes. Additionally, polysaccharides have anti-fatigue properties, thereby improving physical performance and reducing the effects of stress on the body. Essential oils, although they constitute a smaller part of the plant’s chemical composition, also contribute to its therapeutic properties. They contain volatile compounds that may have antibacterial, anti-inflammatory and mood-improving effects. Generally speaking, the biological activity of *S. chinensis* results from the synergistic interaction of various chemical components. The diverse chemical structure of this plant allows it to exert many pharmacological activities, including antioxidant, hepatoprotective, anti-inflammatory, immunomodulatory and anti-fatigue effects. Further research on the relationship between the chemical structure of individual compounds in *S. chinensis* and their biological activity may lead to the development of new therapies for various diseases [89].

## Figures and Tables

**Table 1:** Identification and classification of key active compounds of *S. chinensis*.

Active Compounds of *S. chinensis*	Functional Subgroups (Subclasses)	Example of Compound	Main Activity	Main Location in the Plant	References
**Lignans** polyphenolic substances derived by oxidative coupling of monolignols (most lignans have C_18_ cores, as a dimerisation of C_9_ precursors)	furofuran, furan, dibenzylbutane, dibenzylbutyrolactol, dibenzylbutyrolactones, aryltetralin, darylnaphtalene, ibenzocyclooctadienes	gomisin A, gomisin N	antioxidant, antitumour activity	fruits	[10,11,12,13,14,15,16,17,18,19]
		schisandrin A, γ-schisandrin, and schisandrin C	anti-inflammatory, effects on physical performance and the central nervous system	fruits, leaves	
**Flavonoids** general structure of a C_15_ skeleton that consists of two phenyl rings (A and B) and a heterocyclic ring (C, the ring containing the embedded oxygen)	flavones, flavonols, flavanones, and flavan-3-ols	rutin	antiradical activity, anti-inflammatory	fruits, flowers	[20,21,22,23,24]
		quercetin, isoquercitrin	antiradical activity, anti-inflammatory	fruits, flowers, leaves	
**Phenolic acids** compounds containing a phenol ring and a carboxylic acid residue (C_1_–C_6_)	-	cinnamic acid	antibacterial effect, anti-ageing efffect	leaves	[10,20,25,26]
		chlorogenic acid	antioxidant, normalises blood pressure, regulates blood glucose levels	leaves	
		protocatechuic acids	anti-inflammatory, antihyperglycemic and antiapoptotic activities	fruits	
**Triterpenoids** formed from six isoprene units and share a common C_30_ acyclic precursor, squalene. Different types of ring closure in squalene can give rise to many different types of triterpenoid skeletons; most triterpene skeletons are tetracycles and pentacycles.	cycloartane-type triterpenoids, Schinortriterpenoids	schinchinenlactone	antitumour, anti-inflammatory	leaves, stems	[27,28,29,30,31,32,33]
		wuweizidilactone	neuroprotection	fruits	
**Organic acids** weak acids, they differ in the number of hydroxyl or carboxyl functional groups and C=C bonds in their structures	-	citric acid, malic acid, shikimic acid	antimicrobial activity, benefit the body’s digestion and metabolism	fruits	[34,35,36,37,38]
**Essential oils** a mixture of various chemical compounds, such as ketones, aldehydes, alcohols, esters, lactones, terpenes, amines, thiols; a liquid, volatile odorous substance, found most often in special cells of the secretory tissue of plants	-	ylangene, β-himachalene, α-bergamotene	antibacterial and antiviral effects, immune health support	fruits, seeds	[39,40,41,42,43,44]
